# Supplementary material for: Microglial Heterogeneity and Its Potential Role in Driving Phenotypic Diversity of Alzheimer’s Disease
Source: Int J Mol Sci. 2021 Mar 9;22(5):2780. doi: 10.3390/ijms22052780 (PMC7967159; doi:10.3390/ijms22052780)
Supplement: Supplementary file 1 [file ijms-22-02780-s001.pdf]

Supplementary Materials

Microglial Heterogeneity and Its Potential Role in Driving Phenotypic Diversity of Alzheimer's Disease

Stefano Sorrentino [1,2], Roberto Ascari [3], Emanuela Maderna [2], Marcella Catania [2], Bernardino Ghetti [4], Fabrizio Tagliavini [2], Giorgio Giaccone [2] and Giuseppe Di Fede [2,*]

[1] CNR NANOTEC—Institute of Nanotechnology, 73100 Lecce, Italy; stefano.sorrentino@nanotec.cnr.it
[2] Neurology 5 and Neuropathology Unit, Fondazione IRCCS Istituto Neurologico Carlo Besta, 20133 Milan, Italy; emanuela.maderna@istituto-besta.it (E.M.); marcella.catania@istituto-besta.it (M.C.); giorgio.giaccone@istituto-besta.it (G.G.)
[3] Department of Economics, Management, and Statistics (DEMS)—University of Milano-Bicocca, 20126 Milan, Italy; roberto.ascari@unimib.it
[4] Department of Pathology and Laboratory Medicine, Indiana University, Indianapolis, IN 46202, USA; bghetti@iupui.edu
[5] Scientific Directorate, Fondazione IRCCS Istituto Neurologico Carlo Besta, 20133 Milan, Italy; fabrizio.tagliavini@istituto-besta.it
* Correspondence: giuseppe.difede@istituto-besta.it

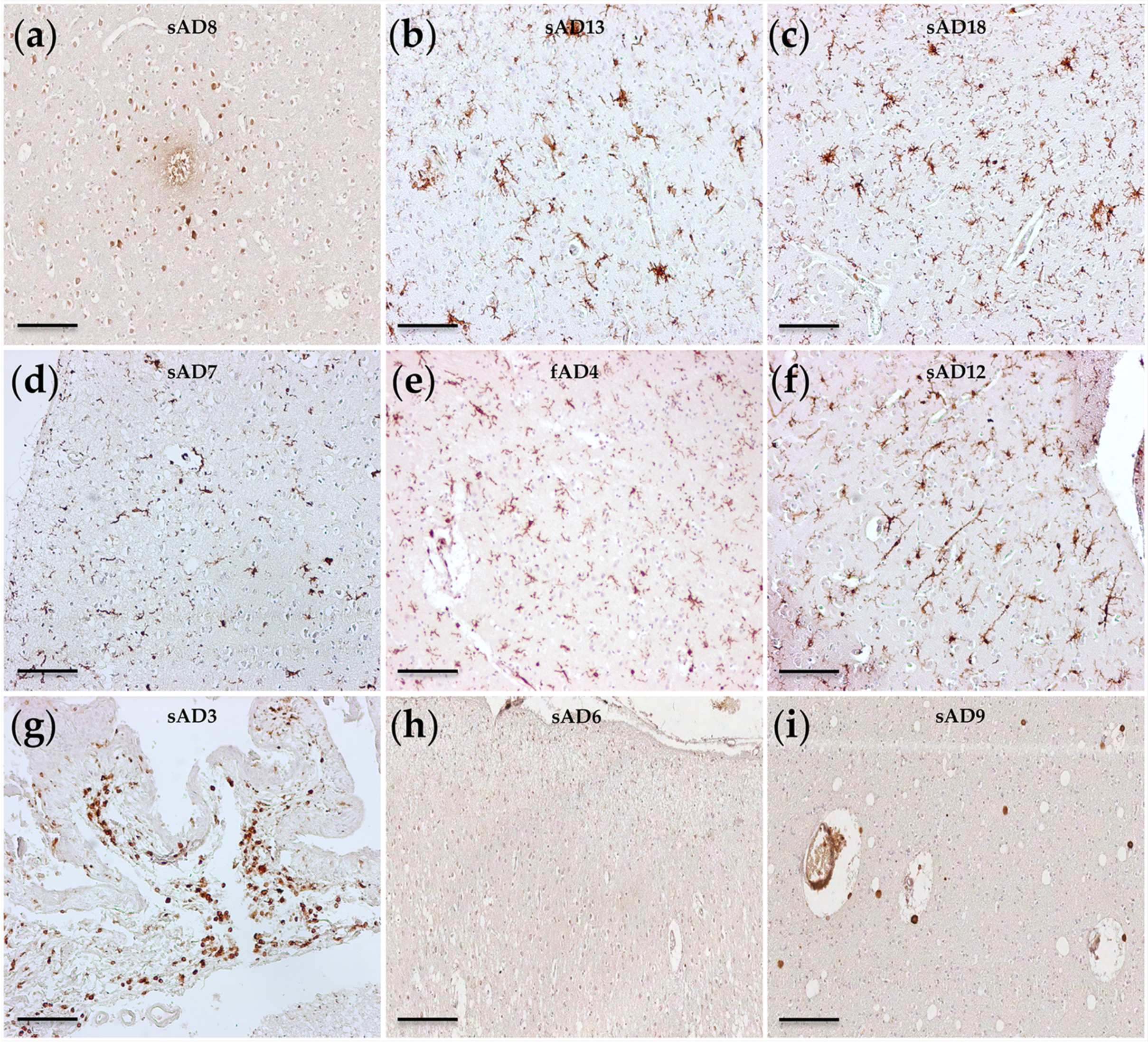

Figure S1. Microglial characterization in AD cases. (**a**) sAD8; (**b**) sAD13; (**c**) sAD18; (**d**) sAD7; (**e**) fAD4 (PS2 A85V); (**f**) sAD12; (**g**) sAD3; (**c**) sAD18; (**h**) sAD6; (**i**) sAD9. Frontal cortex sections immunostained with the IBA1 antibody. Scale bar 50μm.

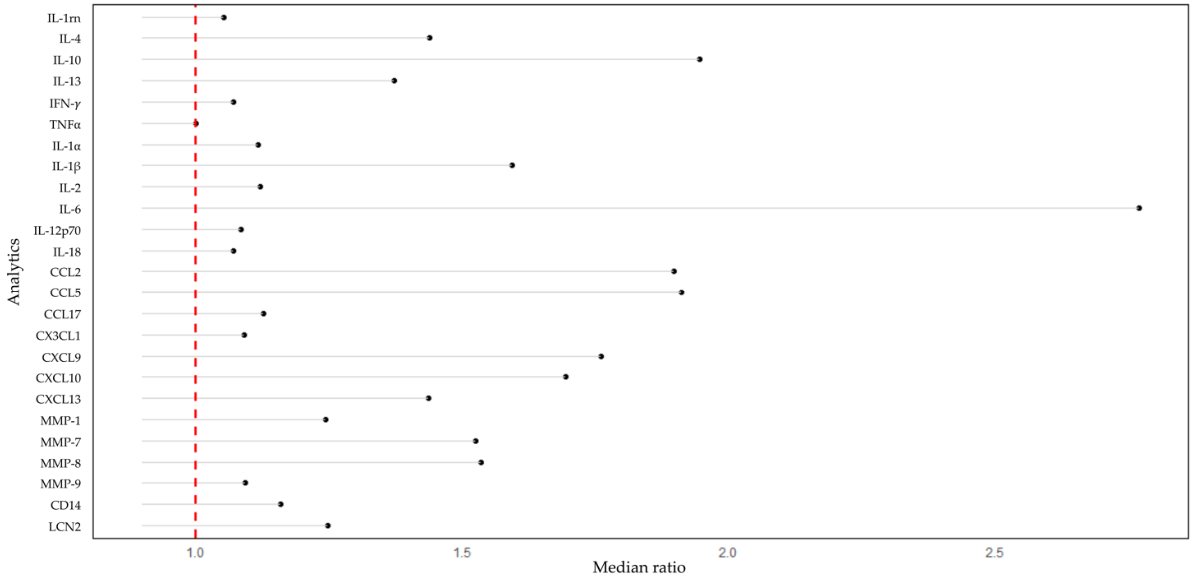

Figure S2. AD Vs CTRLs median (μ1/2) ratio. (ADs μ1/2)/ (CTRLs μ1/2) expresses how much AD samples are elevated respect controls for each cytokine. Ratio = 1 indicates no differences among groups; ratio < 1 indicates that controls are higher than ADs; a ratio > 1 means that AD cases have a higher expression of cytokine respect to controls.

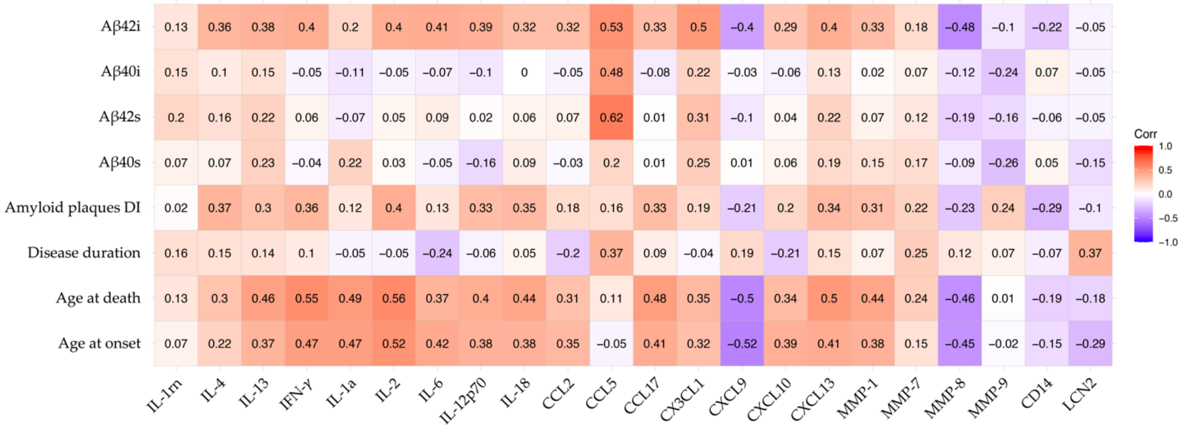

Figure S3. Cytokine association profile. The heatmap shows the coefficient of correlation among cytokines and clinical (age at onset/death, disease duration), neuropathological (Dispersion Index of amyloid deposits: DI) and biochemical (Aβ40 and Aβ42 levels in soluble and insoluble fractions of brain homogenates) data.

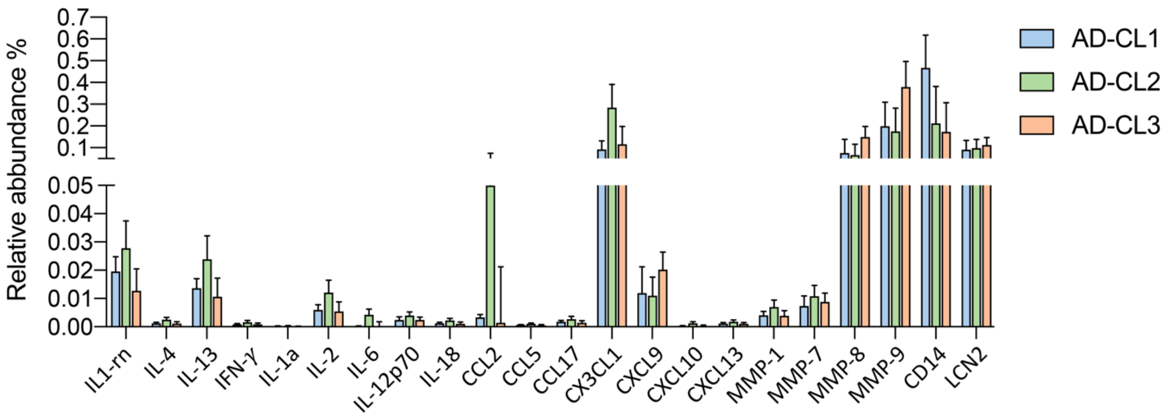

Figure S4. Cytokine profiling. Mean of each cytokine per cluster (AD-CL1=light blue, AD-CL2=green, AD-CL3=orange) represented as percentage on the total amount of neuroinflammatory factors.

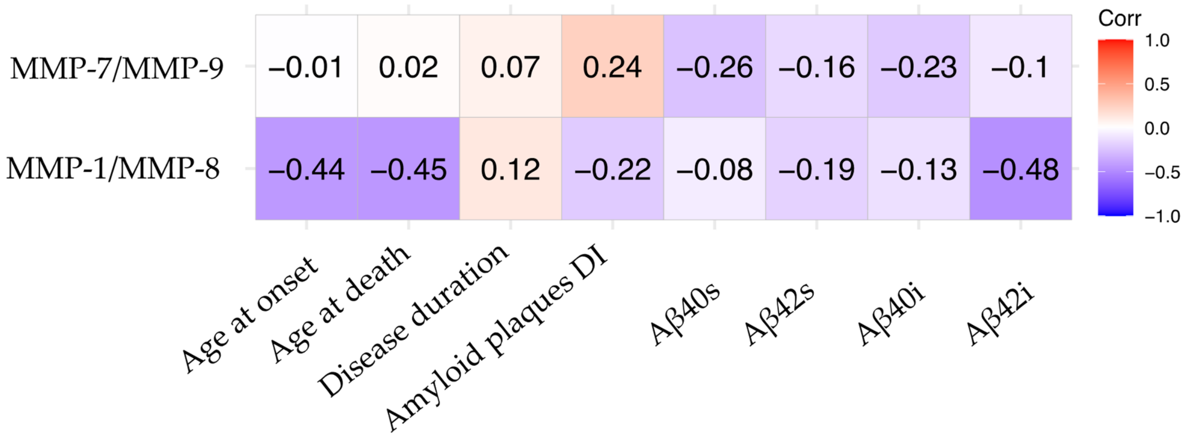

Figure S5. MMPs association profile. The heatmap shows the coefficient of correlation between MMPs subgroups with the following disease indicators: clinical (age at onset/death, disease duration), neuropathological (Dispersion Index of amyloid deposits: DI) and biochemical (Aβ40 and Aβ42 levels in soluble and insoluble fractions of brain homogenates) data.

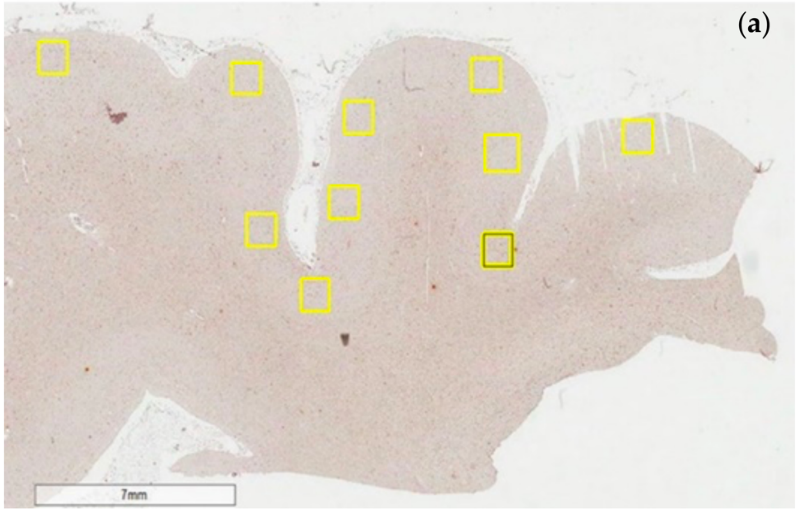

Positive Pixel Count v9
Default Settings

(b)

Input	
View Width	1000
View Height	1000
Overlap Size	0
Image Zoom	1
Markup Compression Type	Same as processed image
Compression Quality	30
Classifier Neighborhood	0
Classifier	None
Class List	
Hue Value	0.1
Hue Width	0.5
Color saturation Threshold	0.04
Iwp(High)	220
Iwp(Low) = Ip (High)	175
Ip(Low) = Isp(High)	100
Isp (Low)	0
Inp(High)	-1

Figure S6. Image analysis procedure. Picture (a) shows a representative IHC with anti-IBA1 antibody to stain microglia in an AD sample. Image was acquired by digital scanner and analysed with image scope software (Leica Biosystems). Yellow squares indicate the Regions Of Interest (ROIs) considered for the analyses. All ROIs were designed with the same dimension and randomly distribute in the grey matter of the frontal cortex. Table in (b) indicate the input parameters chosen to better underline and therefore quantify the microglial content within the ROIs.

Case	Age at onset	Age at death	Disease Duration	Genotype	Braak NFT stage	CAA	Dispersion Index	IBA1		Soluble fraction		Insoluble fraction	
								Number of positive pixel	Intensity of positive pixel	Aβ40	Aβ42	Aβ40	Aβ42
**fAD1**	36	46	10	APP A673V	VI	3	92.05	4195805	311103262	834.6	32.9	277731.1	131.1
**fAD2**	52	57	5	APP A713T	VI	4	123.3	2368319	172109474	167.4	1.3	29718.0	42.2
**fAD3**	36	43	7	PS1 P117A	VI	3	214.5	4800753	352448390	11.5	0.3	6.5	21.8
**fAD4**	60	82	22	PS2 A85V	VI	0	239.3	1749840	138059957	2.3	6.2	26.1	134.4
**sAD1**	79	82	3	sporadic	V	4	96.7	n.a.	n.a.	960.5	3.0	22381.6	66.1
**sAD2**	65	68	3	sporadic	V-VI	1-2	43.55	4121456	280979949	12.5	1.7	115.2	67.5
**sAD3**	n.a.	59	n.a.	sporadic	VI	1-2	143.76	218225	18235990	7.3	0.4	78.8	19.0
**sAD4**	79	81	2	sporadic	III-IV	2-3	108.53	n.a.	n.a.	34.5	4.7	253.5	91.1
**sAD5**	77	83	6	sporadic	VI	3	153.84	n.a.	n.a.	5.9	1.4	10.8	55.1
**sAD6**	60	75	15	sporadic	VI	1	65.21	54433	4762099	6.8	0.9	13.3	8.9
**sAD7**	62	72	10	sporadic	VI	3	240.3	1158578	87430298	22.2	2.5	244.5	92.6
**sAD8**	62	68	6	sporadic	III-IV	2	78.38	60208	5162322	2.2	0.2	221.0	5.1
**sAD9**	83	86	3	sporadic	IV	3	218	108536	8849005	75.9	6.1	3396.2	127.8
**sAD10**	85	90	5	sporadic	III	1	365.57	n.a.	n.a.	2.2	4.3	35.9	129.3
**sAD11**	53	58	5	sporadic	V-VI	2	163.5	n.a.	n.a.	7.7	3.5	81.0	38.1
**sAD12**	50	58	8	sporadic	VI	3	174.4	2995548	230182259	13.4	2.8	19.6	66.7
**sAD13**	58	66	8	sporadic	VI	3	125.3	2175956	172347976	22.4	2.8	15076.5	52.2
**sAD14**	63	69	6	sporadic	VI	3	120	n.a.	n.a.	9.9	2.4	92.4	88.8
**sAD15**	54	62	8	sporadic	VI	2	165.1	3383328	260280908	11.1	3.3	98.1	37.6
**sAD16**	43	47	4	sporadic	V-VI	2-3	146.3	n.a.	n.a.	76.2	2.7	680.0	73.3
**sAD17**	51	59	8	sporadic	V	2	133	1427942	112586626	10.1	3.1	123.1	79.3

**sAD18**	47	61	14	sporadic	VI	3	157.3	1800984	143438752	10.1	2.1	18.5	69.6
**sAD19**	69	72	3	sporadic	V-VI	2	277	n.a.	n.a.	n.a.	n.a.	n.a.	n.a.
**sAD20**	82	86	4	sporadic	IV	3	118.9	n.a.	n.a.	7.2	6.49	321.3	138.0

Table S1. Demographic, clinical, neuropathological and biochemical indicators for the cohort of patients employed in this study. AD = Alzheimer's disease, f = familial. s = sporadic, CAA = cerebral amyloid angiopathy, n.a. = not available. CAA is described by assigning a score ranging from 1 to 4. Amyloid burden is expressed by Dispersion Index (DI) values, as detailed in Methods section of main text. Levels of Aβ40 and Aβ42 in soluble and insoluble fractions of brain homogenates are expressed in ng/g of tissue.

Type	Analyte	Sensitivity (MDD)pg/mL	Standard Curve Range (pg/mL)	Dilution	Sample (μL)
Cytokine	IFN-ɣ	0.4	58.5-14,209	1:1	50
	IL-1α	0.9	5.2-1,270		
	IL-1 rn	18.0	28-6,793		
	IL-1β	0.8	19.5-4,744		
	IL-2	1.8	29.6-7,200		
	IL-4	9.3	14.6-3,550		
	IL-6	1.7	4.8-1,154		
	IL-10	1.6	4.8-1,162		
	IL-12 p70	20.2	137.2-33,340		
	IL-13	32.4	455-110,520		
	IL-18	1.93	54.6-13,260		
	TNF-α	1.2	9.7-2,359		
Chemokine	CCL2/MCP1	9.9	333-8,017	1:1	50
	CCL5/RANTES	1.8	22.6-5,488		
	CCL17/TARC	6.47	85.6-20,790		
	CX3CL1/Fractalkine	64.8	1,114-270,610		
	CXCL9/MIG	18.4	586-142,400		
	CXCL10/IP-10	1.18	2.8-690		
	CXCL13/BLC/BCA-1	11.5	12.6-3,060		
Matrix Metalloproteinase (MMP)	MMP-1	2.7	49.7-12,085	1:1	50
	MMP-7	23.2	350.1-85,082		
	MMP-8	34.2	245.1-59,562		
	MMP-9	13.6	134.1-32,596		
Innate Immunity Factors (IIF)	Lipocalin-2/LCN2	29.2	140-33,920	1:1	50
	CD14	39.6	202-49,180		

Table S2. Samples and reagent preparation. The minimum detection dose (MDD) and the standard curve range are reported for all the analytes tested. Interferon (INF), interleukin (IL), Tumor necrosis factor (TNF), chemokine (C-C motif) ligand (CCL), Chemokine (C-X-C motif) ligand 9 (CXCL), Matrix metalloproteinase (MMP), Cluster of differentiation (CD).
